# Site-Specific Analysis of the Incidence Rate of Enterotoxigenic *Escherichia coli* Infection Elucidates an Association with Childhood Stunting, Wasting, and Being Underweight: A Secondary Analysis of the MAL-ED Birth Cohort

**DOI:** 10.4269/ajtmh.22-0659

**Published:** 2023-04-03

**Authors:** Md Ahshanul Haque, Sabiha Nasrin, Parag Palit, Rina Das, Barbie Zaman Wahid, Md. Amran Gazi, Mustafa Mahfuz, Abu Syed Golam Faruque, Tahmeed Ahmed

**Affiliations:** ^1^ Nutrition and Clinical Services Division, International Centre for Diarrhoeal Disease Research, Bangladesh (icddr,b), Dhaka, Bangladesh;; ^2^ Department of Biostatistics and Epidemiology, School of Public Health and Health Sciences, University of Massachusetts, Amherst, Massachusetts;; ^3^ Gangarosa Department of Environmental Health, Rollins School of Public Health, Emory University, Atlanta, Georgia;; ^4^ University of Nebraska-Lincoln, Lincoln, Nebraska

## Abstract

Asymptomatic infection by fecal enteropathogens is a major contributor to childhood malnutrition. Here, we investigated the incidence rate of asymptomatic infection by enterotoxigenic *Escherichia coli* (ETEC) and assessed its association with childhood stunting, wasting, and being underweight among children under 2 years of age. The Malnutrition and Enteric Disease birth cohort study included 1,715 children who were followed from birth to 24 months of age from eight distinct geographic locations including Bangladesh, Brazil, India, Peru, Tanzania, Pakistan, Nepal, and South Africa. The TaqMan array card assay was used to determine the presence of ETEC in the nondiarrheal stool samples collected from these children. Poisson regression was used to estimate the incidence rate, and multiple generalized estimating equations with binomial family, logit link function, and exchangeable correlation were used to analyze the association between asymptomatic ETEC infection and anthropometric indicators such as stunting, wasting, and being underweight. The site-specific incidence rates of asymptomatic ETEC infections per 100 child-months were also higher at the study locations in Tanzania (54.81 [95% CI: 52.64, 57.07]) and Bangladesh (46.75 [95% CI: 44.75, 48.83]). In the Bangladesh, India, and Tanzania sites, the composite indicator of anthropometric failure was significantly associated with asymptomatic ETEC infection. Furthermore, a significant association between asymptomatic heat-stable toxin ETEC infections and childhood stunting, wasting, and being underweight was found in only the Bangladesh and Tanzania sites.

## INTRODUCTION

In resource-limited settings, being underweight, wasting, and stunting in children are among the most severe public health concerns, and they remain a significant risk factor for death and hinder long-term development. The most common type of chronic malnutrition is stunting, which is defined as a length-for-age *Z* score (LAZ) < −2 standard deviations from the WHO child growth criteria.^1^ The WHO-recommended marker for assessing childhood underweight is the weight-for-age *Z* score (WAZ) < −2.^1^ According to a recent study that pooled data from 62 low- and middle-income countries (LMICs), the overall prevalence of stunting among children under 5 years of age was 29.1%, with 13.7% being underweight.^2^ Wasting is defined by a weight-for-length/height *Z* score (WLZ/WHZ) < −2, and global reports indicate that over 49 million children under the age of 5 suffer from wasting.^1^

Chronic subclinical exposure to fecal enteropathogens as well as the development of environmental enteric dysfunction (EED) is attributed to the underlying pathophysiology of childhood malnutrition. Consequently, childhood diarrhea is a notable risk factor for childhood malnutrition,^3^^,^^4^ whereby diarrheal enteropathogens such as *Campylobacter*, *Shigella*, and enterotoxigenic *Escherichia coli* (ETEC) are associated with poor height and weight gain.^4^^,^^5^ Enterotoxigenic *E. coli* is one of the major enteropathogens that cause moderate-to-severe diarrhea in children under 5 years of age.^6^ Enterotoxigenic *E. coli* is a pathovariant of the gram-negative bacterium *E. coli* and one of the most frequent causes of morbidity and mortality in children in impoverished countries. Infections with ETEC are primarily acquired by the ingestion of fecally contaminated food or water. An earlier study among a malnourished pediatric population from Bangladesh observed an association between ETEC infections and gut health biomarkers, pointing to biological processes that may be involved during these infections and their subsequent influence on malnutrition. Among the enterotoxins of ETEC, heat-stable toxin-ETEC (ST-ETEC) and heat-labile toxin-ETEC (LT-ETEC) cause watery diarrhea.^7^

Findings from another study reported a considerable burden of both ST-ETEC and LT-ETEC in nondiarrheal stool samples of young children in LMICs.^8^ Enteropathogens can be detected directly from stool specimens using very sensitive molecular assays, which have found an association between malnutrition and several common enteropathogens, including LT-ETEC.^9^ In particular, TaqMan array cards provide a very sensitive method for detecting a wide range of enteropathogens.^10^^–^^13^ A study conducted earlier by Rogawski et al. assessed the site-specific prevalence of the asymptomatic ETEC infection and its association with LAZ in children at 2 years of age; it is unclear whether the analysis was longitudinal.^8^ Moreover, the literature also did not investigate the association between all forms of malnutrition (LAZ, WAZ, and WLZ) and the two pathovariants of ETEC (ST-ETEC and LT-ETEC).

In most of the previous studies, the prevalence of stunting, wasting, and being underweight has been reported separately.^14^ In 2000, Peter Svedberg developed a composite indicator of anthropometric failure (CIAF), which provides an overall prevalence of undernutrition (stunting, wasting, or being underweight).^14^ However, studies involving a composite indicator of anthropometric failure are very limited. Consequently, asymptomatic carriage of diarrheal enteropathogens and their subsequent burden among young children have been associated with poor growth.^8^ Enterotoxigenic *E. coli* infection detected by a conventional microbiological culture-based method was found to be prevalent across diverse settings and was associated with growth shortfalls.^6^ The association of ST-ETEC and LT-ETEC infections with stunting, wasting, and being underweight is yet to be addressed. The aim of this paper is to estimate the site-specific incidence rate of ST-ETEC, LT-ETEC, and ETEC infection and assess the association with childhood stunting, wasting, and being underweight, as well as at least one of the growth-faltering symptoms (stunting, wasting, and being underweight) as a CIAF and both stunting and being underweight, among children aged less than 2 years enrolled in the Malnutrition and Enteric Disease (MAL-ED) multicountry birth cohort study.

## MATERIALS AND METHODS

### Study design and participants.

Each site conducted a census of its surrounding area to determine the number of women of reproductive age and the number of children under the age of 5. These statistics were used to determine a catchment region for each site where it was anticipated that > 200 newborns (the goal number of children to be enrolled per site) would be born throughout the course of the 2-year enrollment period.^15^ Study participants were recruited within 17 days of birth from the community in eight distinct geographic locations. After enrolling in the study, the caregivers reported that they had no plans to leave the study’s catchment region for at least 6 months and their willingness to receive twice-weekly home visits. The study sites were Bangladesh (Dhaka), India (Vellore), Nepal (Bhaktapur), Pakistan (Naushero Feroze), South Africa (Venda), Tanzania (Haydom), Brazil (Fortaleza), and Peru (Loreto). Additional inclusion criteria involved maternal age greater than or equal to 16 years, singleton pregnancy cases, and weight at enrollment or birth greater than 1.5 kg.^16^ Furthermore, children with congenital abnormalities or severe neonatal illnesses were excluded from the study. All study sites obtained written informed consent from the parents or caregivers before the enrollment of their children in the study.^15^^,^^16^ All study protocols were carried out following the ethical requirements set by the regulatory authorities at each study location.

### Data collection.

Anthropometric data, as well as information on birth history, household demographics, and maternal factors, were obtained from study participants across all sites at the time of recruitment.^15^ Based on maternal education, improved water and sanitation, eight selected assets, and household income, site-specific composite measures such as the socioeconomic status score and the water/sanitation, assets, maternal education, and income (WAMI) index were established.^17^ Standard guidelines were used to define improved water and sanitation as defined by the WHO.^18^ Filtering, boiling, and the addition of bleaching powder were used to evaluate the treatment of drinking water (Supplemental Table 1). Additionally, data on the children’s birth, household demographics, maternal characteristics, and anthropometric measurements were collected during enrollment. At monthly intervals following enrollment, anthropometric measurements and immunization history were taken.^15^

The anthropometry of the study participants was measured using standard scales (Seca, Hamburg, Germany), and the anthropometric indices of LAZ, WAZ, and WLZ were calculated using the 2006 WHO guidelines for children^9^^,^^19^^,^^20^; children’s growth status can be assessed by combining their sex and age groups using *Z* scores. The WHO *Z*-score scale is linear where the *Z* score = (observed value − average value of the reference population)/standard deviation value of the reference population. For all children of the same age, each interval of the *Z*-score scale corresponds to a set length difference in centimeters.^21^ During biweekly household visits, detailed records of morbidity and child-feeding practices were obtained.^22^ Each month, community research workers collected stool samples across all study locations, which were then conserved and processed using identical and synchronized techniques.^23^

### Outcome variables.

The outcome variables in this analysis were 1) stunting, 2) wasting, 3) being underweight, 4) at least one of them as the CIAF, and 5) both stunting and being underweight. Stunting refers to linear growth faltering. Children were defined as stunted if their LAZ was less than minus two standard deviations (LAZ < −2), wasted if their WLZ was less than minus two standard deviations (WLZ < −2), and being underweight if their WAZ was less than minus two standard deviations (WAZ < −2) of the WHO child growth standards. A study participant’s stunting, wasting, or being underweight was measured as the CIAF.^24^ We also assessed whether a child was both underweight and stunted.

### Laboratory testing.

Our community research staff collected nondiarrheal stool samples monthly. During collection, no fixative was added to the stool samples, and unprocessed stool aliquots were stored in −80 °C freezers before further laboratory testing. The techniques used for laboratory assessments were identical and properly synchronized among all participating laboratories assigned to each of the study sites.^25^^,^^26^ In brief, total nucleic acid extraction from stool samples and subsequent standard optimized protocols were used to detect a total of 29 enteropathogens from a single sample, including ST-ETEC and LT-ETEC, using a customized and compartmentalized probe-based multiplex quantitative polymerase chain reaction system, the TaqMan array card (TAC).^16^^,^^27^ The analytic limit was set at 35 cycles of threshold (Ct), with a Ct value less than 35 being considered positive for all enteropathogens.^9^^,^^28^^,^^29^ In our study, we investigated the occurrence of the LT, STh, and STp genes of ETEC.^29^

### Statistical analysis.

The data were analyzed using Stata software (release 14; StataCorp, College Station, TX). In this study, line graphs were used to examine the prevalence status by time for outcome variables and the variable of asymptomatic ETEC infection. To summarize the data, we used frequency and proportion for qualitative variables, mean and standard deviation for normally distributed quantitative variables, and mean and interquartile range for asymmetric variables. The incidence rate of ETEC infection in all the study sites was estimated using Poisson regression with a log link function, as well as the incidence rate ratio. The number of ETEC infections during 0–24 months was defined as the outcome variable of Poisson regression, and log(number of stool examinations) was defined as the offset variable. The Poisson null model was used to estimate the site-specific incidence rate. The incidence rate ratios of Brazil, India, Nepal, Peru, Pakistan, South Africa, and Tanzania when compared with the Bangladesh site were estimated using multiple Poisson regression, where the independent variable was site. The child’s sex, WAMI index, maternal height, and mother having fewer than three living children were adjusted in the multiple Poisson regression model.

Multiple generalized estimating equations (GEEs) with binomial family, logit link function, and exchangeable correlation were used to analyze the association between asymptomatic ETEC infection and anthropometric indicators. In the GEE, we adjusted for the WAMI index, child’s sex, maternal height, mother having fewer than three live children, and *Campylobacter jejuni/coli*, enteroaggregative *Escherichia coli* (EAEC), and *Shigella*/enteroinvasive *Escherichia coli* (EIEC) as copathogen infections based on the literature review as well as our bivariate analyses.^17^ The age of the participants was used as a time variable in the model as a result of the birth cohort study. In addition, the adjusted odds ratio was estimated as the strength of association to explain the regression model, and a 95% confidence interval was used to denote significance level.

## RESULTS

### General characteristics.

A total of 1,715 participants, who completed follow-ups for 24 months, contributed 34,622 surveillance stool samples tested for the identification of ETEC. The demographic characteristics of the study participants from all the studies are presented in Table 1. Wasting and being underweight were not included (*N* = 165) for the Brazil site (Fortaleza) in the GEE analysis because of the very low prevalence of those statuses. Stunting and wasting were not included (*N* = 246) for the Pakistan site (Naushero Feroze) in the GEE analysis because of unavailable childhood length data.

**Table 1:** General characteristics of the study subjects (*N* = 1,715)

Characteristics	Bangladesh (*N* = 210)	Brazil (*N* = 165)	India (*N* = 227)	Nepal (*N* = 227)	Peru (*N* = 194)	Pakistan (*N* = 246)	South Africa (*N* = 237)	Tanzania (*N* = 209)	Overall (*N* = 1,715)
Male sex, *n* (%)	108 (51.4)	89 (53.9)	105 (46.3)	122 (53.7)	105 (54.1)	120 (48.8)	120 (50.6)	105 (50.2)	874 (51.0)
Days of exclusive breastfeeding*	155 (116, 176)	81 (48, 132)	107 (72, 140)	86 (44, 135)	87 (33, 146)	14 (8, 19)	31 (18, 52)	58 (36, 81)	67 (28, 124)
Birth weight (kg), mean ± SD	2.8 ± 0.4	3.4 ± 0.5	2.9 ± 0.4	3 ± 0.4	3.1 ± 0.4	2.7 ± 0.4	3.2 ± 0.5	3.2 ± 0.5	3.0 ± 0.5
Weight-for-age *Z* score at enrollment, mean ± SD	−1.3 ± 0.9	−0.2 ± 1.0	−1.3 ± 1.0	−0.9 ± 1.0	−0.6 ± 0.9	−1.4 ± 1.0	−0.4 ± 1.0	−0.1 ± 0.9	−0.8 ± 1.1
Length-for-age *Z* score at enrollment, mean ± SD	−0.96 ± 1.0	−0.8 ± 1.1	−1.0 ± 1.1	−0.7 ± 1	−0.9 ± 1	−1.3 ± 1.1	−0.7 ± 1.0.	−1 ± 1.1	−0.9 ± 1.1
Length-for-age *Z* score at 24 months, mean ± SD	−2.0 ± 0.9	0 ± 1.1	−1.9 ± 1	−1.3 ± 0.9	−1.9 ± 0.9	N/A	−1.7 ± 1.1	−2.7 ± 1	−1.7 ± 1.2
Maternal age (years), mean ± SD	25.0 ± 5.0	25.4 ± 5.6	23.9 ± 4.2	26.6 ± 3.7	24.8 ± 6.3	28.1 ± 5.9	27 ± 7.2	29.1 ± 6.5	26.3 ± 5.9
Maternal weight (kg), mean ± SD	49.7 ± 8.5	62 ± 11.5	50.3 ± 9.3	56.2 ± 8.3	56.3 ± 9.6	50.7 ± 9.6	68 ± 15.3	55.7 ± 8.8	55.9 ± 12
Maternal height (cm), mean ± SD	149.0 ± 5.0	155.1 ± 6.7	151.1 ± 5.2	149.7 ± 5.3	150.2 ± 5.5	153.4 ± 5.7	158.7 ± 6.6	155.9 ± 5.9	152.9 ± 6.6
Maternal educational level < 6 years, *n* (%)	133 (63.3)	22 (13.3)	80 (35.2)	59 (26)	44 (22.7)	202 (82.1)	5 (2.1)	75 (35.9)	620 (36.2)
Mother has fewer than 3 living children, *n* (%)	160 (76.2)	113 (68.5)	157 (69.8)	199 (87.7)	111 (57.2)	105 (42.7)	141 (59.5)	58 (27.8)	1044 (61)
Ownership of chickens/ducks, *n* (%)	3 (1.4)	1 (0.6)	14 (6.2)	73 (32.2)	75 (38.7)	144 (62.3)	87 (37.2)	204 (97.6)	601 (35.4)
Ownership of cows/bulls, *n* (%)	1 (0.5)	–	5 (2.2)	3 (1.3)	–	146 (59.4)	33 (13.9)	157 (75.1)	345 (20.1)
Routine treatment of drinking water, *n* (%)	130 (61.9)	10 (6.1)	7 (3.1)	98 (43.2)	32 (16.5)	–	12 (5.1)	12 (5.7)	301 (17.6)
Improved drinking water source, *n* (%)	210 (100)	165 (100)	227 (100)	227 (100)	184 (94.9)	246 (100)	196 (82.7)	89 (42.6)	1544 (90.0)
Improved latrine, *n* (%)	210 (100)	165 (100)	121 (53.3)	227 (100)	66 (34)	197 (80.1)	232 (97.9)	19 (9.1)	1237 (72.1)
Improved floor, *n* (%)	204 (97.1)	165 (100)	222 (97.8)	109 (48)	69 (35.6)	81 (32.9)	231 (97.5)	13 (6.2)	1094 (63.8)
Monthly income < $150, *n* (%)	69 (32.9)	161 (97.6)	19 (8.4)	106 (46.7)	58 (29.9)	115 (46.8)	179 (75.5)	0 (0)	707 (41.2)
WAMI score, mean ± SD	0.6 ± 0.1	0.8 ± 0.1	0.5 ± 0.2	0.7 ± 0.1	0.5 ± 0.1	0.5 ± 0.2	0.8 ± 0.1	0.2 ± 0.1	0.6 ± 0.2

N/A = not available; WAMI = water/sanitation, assets, maternal education, and income.

* Median (Q1, Q3), where Q1 and Q3 are the first and third quartile.

**Figure 1.**
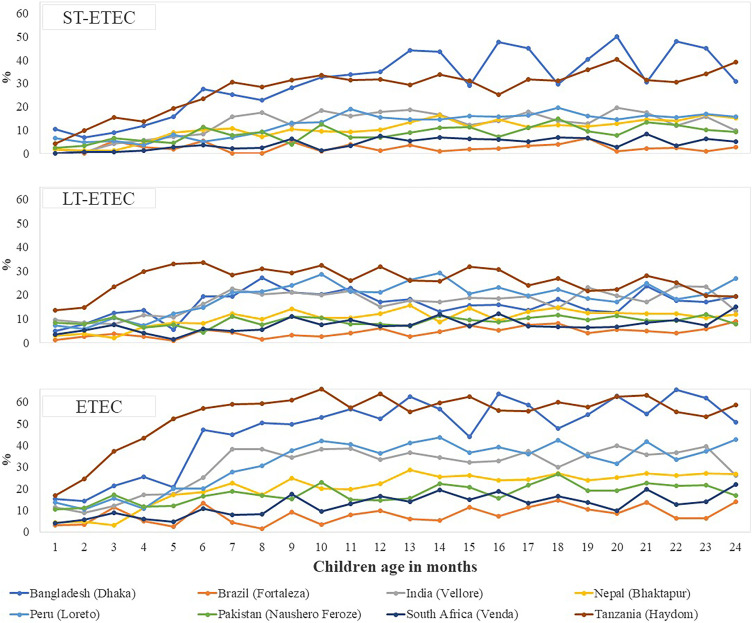
Site-specific prevalence of enterotoxigenic *Escherichia coli* (ETEC), heat-stable toxin-ETEC (ST-ETEC), and heat-labile toxin-ETEC (LT-ETEC) infection by children’s ages in months.

**Figure 2.**
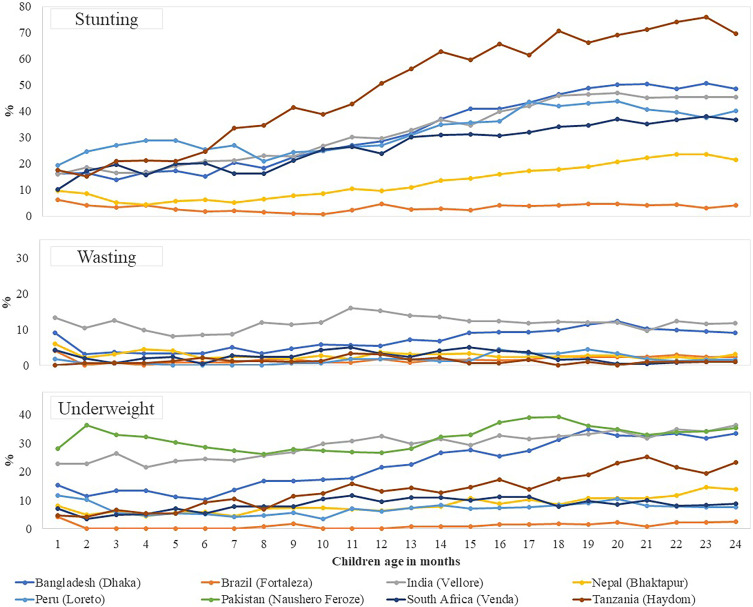
Site-specific prevalence of stunting, wasting, and being underweight by children’s ages in months. Stunting and wasting were not included for the Pakistan site (Naushero Feroze) in this figure analysis because of unavailable childhood length.

The site-specific prevalence of asymptomatic ETEC infection by follow-up is presented in Figure 1. Compared with overall infection, the prevalence of asymptomatic ETEC infections was higher in Bangladesh and Tanzania than in the other sites. At each of the time points when samples were taken, the Brazil site had the lowest prevalence. The site-specific prevalence of stunting, wasting, and being underweight is given in Figure 2. When compared between the sites, the prevalence of stunting increased over time and was higher in Bangladesh and Tanzania. The site-specific prevalence of *C. jejuni/coli*, EAEC, and *Shigella*/EIEC infection by children aged in months has been presented in Supplemental Figure 1.

### Incidence rate of ETEC infection.

Site-specific incidence rates per 100 child-months and incidence rate ratios for all study sites for asymptomatic infections by ST-ETEC, LT-ETEC, and ETEC when compared with Bangladesh have been given in Table 2. Compared with Bangladesh (incidence rate [IR]: 30.46 [95% CI: 28.86, 32.15]), the incidence rates of asymptomatic ST-ETEC infections were lower in all other sites. Moreover, the incidence rates of asymptomatic LT-ETEC infections were higher in Peru (IR: 19.28 [95% CI: 18.00, 20.66]) and Tanzania (IR: 26.34 [95% CI: 24.84, 27.93]) than in Bangladesh (IR: 16.19 [95% CI: 15.03, 17.44]). As for ETEC infection, the incidence rates were significantly lower in all sites compared with Bangladesh, except for Tanzania, where the incidence rate was insignificant.

**Table 2:** Site-specific incidence rate and incidence rate ratio compared with Bangladesh

Type of ETEC and locations	Incidence rate per 100 child-months (95% CI)*	Adjusted incidence rate ratio (95% CI)†	*P* value
ST-ETEC			
Bangladesh (Dhaka)	30.46 (28.86, 32.15)	Reference	–
Brazil (Fortaleza)	2.36 (1.86, 3.00)	0.09 (0.07, 0.11)	< 0.001
India (Vellore)	12.84 (11.86, 13.90)	0.41 (0.37, 0.45)	< 0.001
Nepal (Bhaktapur)	10.44 (9.59, 11.37)	0.37 (0.33, 0.41)	< 0.001
Peru (Loreto)	12.61 (11.58, 13.73)	0.41 (0.37, 0.45)	< 0.001
Pakistan (Naushero Feroze)	8.67 (7.86, 9.56)	0.27 (0.24, 0.31)	< 0.001
South Africa (Venda)	4.09 (3.54, 4.72)	0.15 (0.13, 0.18)	< 0.001
Tanzania (Haydom)	28.27 (26.72, 29.92)	0.79 (0.70, 0.89)	< 0.001
LT-ETEC			
Bangladesh (Dhaka)	16.19 (15.03, 17.44)	Reference	–
Brazil (Fortaleza)	4.33 (3.63, 5.17)	0.28 (0.23, 0.34)	< 0.001
India (Vellore)	17.18 (16.04, 18.40)	1.07 (0.96, 1.18)	0.229
Nepal (Bhaktapur)	10.42 (9.57, 11.35)	0.66 (0.58, 0.74)	< 0.001
Peru (Loreto)	19.28 (18.00, 20.66)	1.19 (1.08, 1.32)	< 0.001
Pakistan (Naushero Feroze)	9.10 (8.27, 10.01)	0.57 (0.50, 0.64)	< 0.001
South Africa (Venda)	7.40 (6.66, 8.24)	0.49 (0.42, 0.56)	< 0.001
Tanzania (Haydom)	26.34 (24.84, 27.93)	1.60 (1.41, 1.82)	< 0.001
ETEC			
Bangladesh (Dhaka)	46.75 (44.75, 48.83)	Reference	–
Brazil (Fortaleza)	7.73 (6.78, 8.82)	0.18 (0.16, 0.21)	< 0.001
India (Vellore)	30.66 (29.13, 32.27)	0.65 (0.60, 0.69)	< 0.001
Nepal (Bhaktapur)	20.92 (19.7, 22.22)	0.47 (0.43, 0.51)	< 0.001
Peru (Loreto)	31.99 (30.33, 33.74)	0.68 (0.64, 0.73)	< 0.001
Pakistan (Naushero Feroze)	17.85 (16.68, 19.11)	0.38 (0.35, 0.41)	< 0.001
South Africa (Venda)	12.55 (11.56, 13.61)	0.29 (0.26, 0.32)	< 0.001
Tanzania (Haydom)	54.81 (52.64, 57.07)	1.07 (0.98, 1.17)	0.118

ETEC = enterotoxigenic *Escherichia coli*; LT-ETEC = heat-labile-ETEC; ST-ETEC = heat-stable toxin-ETEC.

* Incidence rates (95% CI) were calculated with the Poisson null model.

† Adjusted for sex, WAMI (water/sanitation, assets, maternal education, and income) index, maternal height, and mother has fewer than three living children in the multiple Poisson regression model.

### Association between ETEC infection and childhood malnutrition.

Asymptomatic ST-ETEC infection as denoted by detection of ST-ETEC from nondiarrheal stools by TAC was found to be associated with stunting (odds ratio [OR]: 1.26 [95% CI: 1.19, 1.34]), wasting (OR: 1.20 [95% CI: 1.05, 1.36]), being underweight (OR: 1.18 [95% CI: 1.11, 1.25]), and CIAF (OR: 1.22 [95% CI: 1.16, 1.29]). However, the detection of LT-ETEC was not associated with any form of childhood malnutrition.

On the other hand, asymptomatic ETEC infection (LT-ETEC, ST-ETEC, or both) was associated with stunting (OR: 1.20 [95% CI: 1.15, 1.26]), wasting (OR: 1.19 [95% CI: 1.07, 1.32]), being underweight (OR: 1.16 [95% CI: 1.11, 1.22]), and CIAF (OR: 1.17 [95% CI: 1.12, 1.22]). However, if we look at the site-specific strength of associations, only in the Bangladesh, India, and Tanzania sites was CIAF significantly associated with asymptomatic ETEC infection. Consequently, LT-ETEC was significantly associated with stunting only in India (OR: 1.13 [95% CI: 1.01, 1.27]) and South Africa (OR: 1.22 [95% CI: 1.02, 1.46]). Consequently, LT-ETEC was found to be significantly associated with wasting only in India (OR: 1.20 [95% CI: 1.01, 1.42]). If we consider the children with both stunting and being underweight, then the indicator was associated only with the Bangladesh and Tanzania sites. The site-specific strengths of association between ST-ETEC, LT-ETEC, and ETEC infection and nutritional status of the study participants have been presented in Table 3.

**Table 3:** Site-specific strength of association between enterotoxigenic *Escherichia coli* infection and child’s nutritional status

Indicator and location	ST-ETEC		LT-ETEC		ETEC	
	Adjusted OR (95% CI)	*P* value	Adjusted OR (95% CI)	*P* value	Adjusted OR (95% CI)	*P* value
Stunting (LAZ < −2)						
Bangladesh (Dhaka)	1.24 (1.11, 1.38)	0.000	1.06 (0.93, 1.22)	0.377	1.25 (1.12, 1.38)	0.000
Brazil (Fortaleza)*	1.92 (0.69, 5.32)	0.209	0.31 (0.08, 1.31)	0.112	0.68 (0.29, 1.61)	0.382
India (Vellore)	1.21 (1.06, 1.38)	0.006	1.14 (1.01, 1.28)	0.029	1.21 (1.10, 1.34)	0.000
Nepal (Bhaktapur)	0.91 (0.74, 1.13)	0.401	1.03 (0.84, 1.26)	0.772	0.97 (0.83, 1.13)	0.693
Peru (Loreto)	1.05 (0.90, 1.22)	0.570	0.98 (0.86, 1.11)	0.707	1.00 (0.90, 1.12)	0.939
South Africa (Venda)	1.05 (0.82, 1.35)	0.697	1.22 (1.02, 1.46)	0.034	1.17 (1.00, 1.36)	0.046
Tanzania (Haydom)	1.43 (1.28, 1.61)	0.000	0.98 (0.87, 1.10)	0.686	1.33 (1.20, 1.48)	0.000
Wasting (WLZ < −2)						
Bangladesh (Dhaka)	1.07 (0.87, 1.33)	0.513	0.92 (0.70, 1.20)	0.538	1.02 (0.83, 1.24)	0.857
India (Vellore)	1.05 (0.86, 1.28)	0.621	1.20 (1.01, 1.42)	0.034	1.17 (1.01, 1.35)	0.035
Nepal (Bhaktapur)	1.43 (0.91, 2.24)	0.118	0.92 (0.56, 1.53)	0.758	1.18 (0.82, 1.71)	0.367
Peru (Loreto)	0.90 (0.45, 1.83)	0.780	1.14 (0.66, 1.97)	0.641	1.04 (0.65, 1.69)	0.860
South Africa (Venda)	1.80 (0.93, 3.48)	0.080	1.32 (0.76, 2.27)	0.322	1.53 (0.98, 2.39)	0.061
Tanzania (Haydom)	2.36 (1.33, 4.19)	0.003	0.82 (0.43, 1.58)	0.561	2.14 (1.12, 4.09)	0.021
Being underweight (WAZ < −2)						
Bangladesh (Dhaka)	1.10 (0.98, 1.24)	0.120	1.10 (0.95, 1.26)	0.204	1.15 (1.03, 1.29)	0.014
India (Vellore)	1.11 (0.98, 1.26)	0.110	1.06 (0.95, 1.19)	0.297	1.10 (1.00, 1.21)	0.042
Nepal (Bhaktapur)	1.10 (0.87, 1.40)	0.415	0.93 (0.73, 1.20)	0.593	1.02 (0.84, 1.22)	0.872
Peru (Loreto)	1.07 (0.82, 1.39)	0.620	1.02 (0.82, 1.27)	0.860	1.05 (0.87, 1.27)	0.618
Pakistan (Naushero Feroze)†	1.14 (0.98, 1.34)	0.099	1.03 (0.88, 1.21)	0.734	1.09 (0.97, 1.23)	0.143
South Africa (Venda)	1.19 (0.83, 1.72)	0.349	0.84 (0.63, 1.13)	0.258	0.96 (0.76, 1.22)	0.747
Tanzania (Haydom)	1.27 (1.08, 1.48)	0.003	1.07 (0.91, 1.26)	0.424	1.33 (1.14, 1.55)	0.000
CIAF (LAZ < −2, WLZ < −2, or WAZ < −2)						
Bangladesh (Dhaka)	1.17 (1.06, 1.30)	0.003	1.07 (0.94, 1.21)	0.320	1.19 (1.08, 1.31)	0.000
Brazil (Fortaleza)*	1.70 (0.80, 3.61)	0.170	0.31 (0.10, 0.95)	0.040	0.67 (0.36, 1.26)	0.211
India (Vellore)	1.13 (0.99, 1.27)	0.064	1.10 (0.99, 1.23)	0.079	1.14 (1.04, 1.25)	0.005
Nepal (Bhaktapur)	0.98 (0.81, 1.19)	0.842	0.99 (0.82, 1.20)	0.920	0.98 (0.85, 1.14)	0.819
Peru (Loreto)	1.02 (0.88, 1.19)	0.790	0.95 (0.84, 1.08)	0.470	0.98 (0.87, 1.09)	0.663
South Africa (Venda)	1.00 (0.78, 1.29)	0.973	1.18 (0.99, 1.41)	0.069	1.12 (0.97, 1.31)	0.129
Tanzania (Haydom)	1.45 (1.30, 1.63)	0.000	1.00 (0.89, 1.12)	0.947	1.37 (1.23, 1.52)	0.000
Stunting and being underweight only (LAZ < −2 and WAZ < −2)						
Bangladesh (Dhaka)	1.16 (1.01, 1.34)	0.039	1.08 (0.91, 1.28)	0.396	1.19 (1.04, 1.37)	0.011
India (Vellore)	1.17 (1.00, 1.36)	0.045	1.11 (0.97, 1.27)	0.126	1.17 (1.05, 1.31)	0.006
Nepal (Bhaktapur)	1.05 (0.79, 1.40)	0.719	1.02 (0.77, 1.35)	0.907	1.04 (0.84, 1.29)	0.720
Peru (Loreto)	1.13 (0.85, 1.50)	0.389	1.06 (0.84, 1.35)	0.603	1.12 (0.91, 1.37)	0.294
South Africa (Venda)	1.36 (0.92, 2.03)	0.125	0.86 (0.61, 1.19)	0.357	1.03 (0.79, 1.34)	0.833
Tanzania (Haydom)	1.26 (1.07, 1.48)	0.005	1.02 (0.87, 1.21)	0.793	1.28 (1.09, 1.49)	0.002

CIAF = composite indicator of anthropometric failure; EAEC = enteroaggregative *Escherichia coli*; EIEC = enteroinvasive *Escherichia coli*; ETEC = enterotoxigenic *Escherichia coli*; LAZ = length-for-age *Z* score; LT-ETEC = heat-labile toxin-ETEC; ST-ETEC = heat-stable toxin-ETEC; WAZ = weight-for-age *Z* score; WLZ = weight-for-length *Z* score. Adjusted in the generalized estimating equation (GEE) for sex, WAMI (water/sanitation, assets, maternal education, and income) index, maternal height, mother has fewer than three living children, and *Campylobacter jejuni/coli*, EAEC, and *Shigella*/EIEC as copathogen infection. Dependent variable: stunting (LAZ < −2), wasting (WLZ < −2), being underweight (WAZ < −2), CIAF (LAZ < −2, WLZ < −2, or WAZ < −2), and stunting and being underweight only (LAZ < −2 and WAZ < −2). Independent variables: ETEC infection.

* Wasting and being underweight were not included for the Brazil site (Fortaleza) in the GEE analysis as a result of very small prevalence.

† Stunting and wasting were not included for the Pakistan site (Naushero Feroze) in the GEE analysis as a result of unavailable childhood length.

## DISCUSSION

The current study documented the incidence rate of asymptomatic infection by the distinct virulent strains of ETEC, namely ST-ETEC and LT-ETEC, and assessed their association with the various forms of childhood malnutrition as well as the CIAF among children enrolled in the MAL-ED birth cohort study. The incidence rate of asymptomatic ETEC infection varied significantly among sites. Our study revealed that the incidence rate per 100 child-months was significantly higher in Bangladesh and Tanzania than in India, Nepal, Peru, and South Africa. Moreover, the prevalence of stunting in the Tanzania and Bangladesh sites over time was higher than in the other sites. In our site-specific association analysis, only the Bangladesh and Tanzania sites had a significant association between ST-ETEC and almost all forms of childhood malnutrition, whereas in India, only an association between ST-ETEC and childhood stunting was reported. Malnutrition has been linked to enteric dysfunction and subsequent gut inflammation, which may indicate a possible association between malnutrition and ST-ETEC prevalence. Another study found ST-ETEC to have a strong association with diarrhea among children with acute malnutrition, which is also consistent with our findings.^30^

Based on LT-ETEC infection, site-specific analysis depicted a significant association of LT-ETEC with stunting and wasting for India, whereas South Africa exhibited a significant association only with stunting. When both stunting and being underweight in children were considered, this indicator had a strong association with ST-ETEC and overall ETEC asymptomatic infection but not in the case of LT-ETEC. When prior studies conducted in Bangladesh are considered, several previously published articles reported that ETEC infection hindered childhood growth.^31^^–^^33^

Enterotoxigenic *Escherichia coli* produces two toxins that cause watery diarrhea: the heat-stable toxin and the heat-labile toxin. The nutritional status of the children was found to be associated with ST-ETEC diarrhea. Thus far, children who have had one or more bouts of diarrhea as a result of ST-ETEC infection are substantially malnourished and have stunted growth by the age of 2.^34^ This is substantiated by previous findings that have found that ETEC diarrheal incidents in childhood can have a detrimental impact on children’s growth.^5^ Reports from a study conducted in Bangladesh showed that children with ETEC diarrhea were underweight and had delayed growth during a follow-up period of 2 years. Consequently, a birth cohort study in Bangladesh also found that children who were malnourished at birth had increased ETEC infections and more severe diarrhea compared with children who were not malnourished at birth.^33^

The heat-stable enterotoxin secreted by ST-ETEC is a cysteine-rich peptide. It binds to guanylate cyclase C on the surface of intestinal epithelia, resulting in the generation of cyclic guanosine monophosphate, which leads to salt and water accumulation in the intestinal lumen and watery diarrhea.^35^^,^^36^ A previous study conducted in Bangladesh on children from 0 to 5 years of age showed that of total ETEC isolates, 49.4% were positive for ST-producing ETEC and 25.4% were positive for LT-producing ETEC. According to this study’s findings, ETEC isolates surged in the sweltering summer and sharply fell in the winter.^36^ ETEC in Bangladesh has previously been shown to follow a very distinctive biennial seasonality with two distinct peaks, one at the start of summer and the other in the fall months.^35^ Tanzania also has hot summers; hence, temperatures could be a reason behind the similarity of ST-ETEC prevalence in Bangladesh and Tanzania. Earlier epidemiological data suggest that strains of ETEC secreting ST, with or without LT, induce the most severe disease among children in developing countries.^37^^,^^38^ In addition, ST is present in approximately 75% of all clinical ETEC isolates and seems to be associated with more severe illness than LT. We hypothesize that this may be one of the underlying causes of asymptomatic ST-ETEC infections being associated with childhood stunting, wasting, and being underweight in our study.

As mentioned before, the incidence rate of ETEC per 100 child-months was the highest in the Bangladesh and Tanzania sites, which explains the significant site-specific association of ST-ETEC with malnutrition. In India and South Africa, on the other hand, LT-ETEC has been associated with impaired growth,^39^^,^^40^ and heat-labile toxin has been recommended as a marker for the presence of particular factors associated with colonization that may influence the association with malnutrition. Tropical sprue, also known as tropical enteropathy or EED, usually occurs alongside diarrheal diseases in young children in developing nations, impairing their cognitive development, growth, and responsiveness to oral immunizations.^35^ Malnutrition or kwashiorkor has long been observed in developing nations following an acute diarrheal infection. According to cohort studies, children in highly endemic parts of Bangladesh were more prone to being underweight and/or stunted after diarrhea induced by ETEC.^35^ Malnourished children, on the other hand, appear to be at a greater risk of diarrheal illness and the development of ETEC-related protracted diarrhea.^35^ Additionally, earlier studies revealed that EPEC and EHEC, pathovariants of *E. coli*, attach to intestinal cells and use the type III secretion system (T3SS), a syringe-like molecular machinery, to inject at least 25 different bacterial effector proteins into the host cell. These effectors regulate a wide spectrum of host signaling pathways, including inflammasome pathways that once translocated into mammalian cells, disrupting host cell function and promoting virulence.^41^^,^^42^ Hence, there might be a possibility that ETEC, another pathovariant of *E. coli*, also utilizes the T3SS to regulate inflammasome pathways in mammalian cells, which may subsequently promote intestinal inflammation.

### Limitations.

Because of its birth cohort design, there were no records of pre-event pathogen infection, and thus we were unable to establish any potential causal relationship between the pathogen burden and the outcome variables. We have addressed only three copathogens. Therefore, a major limitation was that this study did not address the presence of other pathogens either as possible coinfections, previous infections within children, or the overall burden of other enteric pathogens in each site’s population/environment. The *Z*-score values were converted into binary indicators, for which we had to lose some information.

## CONCLUSION

In LMICs around the world, ETEC is a common cause of diarrheal disease, posing a particular threat to the health of young children. Moreover, acute malnutrition paired with diarrhea caused by ETEC puts children at a high risk of death. Our study aimed to investigate a possible association between specific pathogenic variants of ETEC, ST-ETEC, and LT-ETEC with the CIAF, detected from nondiarrheal stool samples. Our findings demonstrate that the incidence rate of asymptomatic ETEC infections was higher in the Bangladesh and Tanzania sites when compared with other sites. Moreover, our site-specific analysis revealed a significant association between ST-ETEC and childhood stunting, wasting, and being underweight in the Bangladesh and Tanzania sites. The results of our study highlight the clinical importance of ETEC infection by addressing the long-term effects caused by asymptomatic gut colonization of ETEC in young children. This may prompt increased clinical supervision and monitoring of ETEC infections in early childhood to prevent the long-term complications that follow on a larger scale.

## Financial Disclosure

This work was supported, in whole or in part, by The Bill & Melinda Gates Foundation (grant OPP47075). Under the grant conditions of the foundation, a Creative Commons Attribution 4.0 Generic License has already been assigned to the Author’s Accepted Manuscript version that might arise from this submission.

## Supplemental Materials


Supplemental materials
