# Supplementary material for: Site-Specific Analysis of the Incidence Rate of Enterotoxigenic Escherichia coli Infection Elucidates an Association with Childhood Stunting, Wasting, and Being Underweight: A Secondary Analysis of the MAL-ED Birth Cohort
Source: Am J Trop Med Hyg. 2023 Apr 3;108(6):1192–200. doi: 10.4269/ajtmh.22-0659 (PMC10540109; doi:10.4269/ajtmh.22-0659)
Supplement: Supplementary file 1 [file tpmd220659.SD1.pdf]

Supplementary Table 1. Water/sanitation, Assets, Maternal education, and Income (WAMI) index calculation

Indicators	Description	Score
Water/sanitation	Improved Water=4<br>Improved sanitation=4	4*2=8
Assets	Eight selected assets: Separate room for a kitchen, Household bank account, Mattress, Refrigerator, People per room≥2, Table, Chair or bench	8*1=8
Maternal education	Mother provided the number of years of schooling (0-16y)	16/2=8
Income	Monthly household income was converted to US dollars using the exchange rate from January 1, 2010. Income was divided into octiles using the following scores and cutoffs: 1 (0–26), 2 (26.01-47), 3 (47.01-72), 4 (72.01-106), 5 (106.01-135), 6 (135.01-200), 7 (200.01-293), 8 (293+)	8
Total		32
Then the WAMI index = the total number of observed score /32		

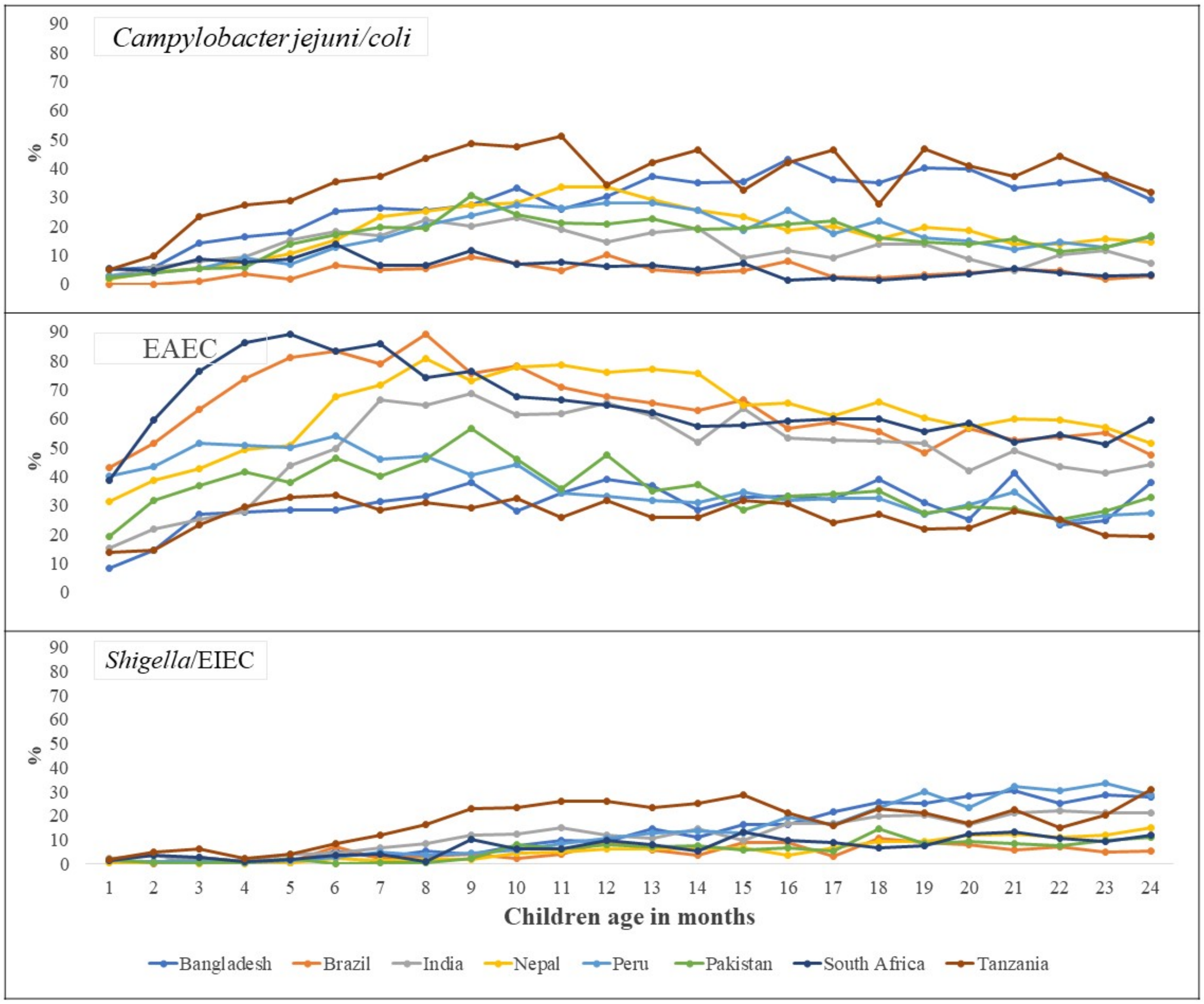

Supplementary Figure 1. Site specific prevalence of *Campylobacter jejuni/coli*, EAEC and *Shigella*/EIEC infection by children age in months

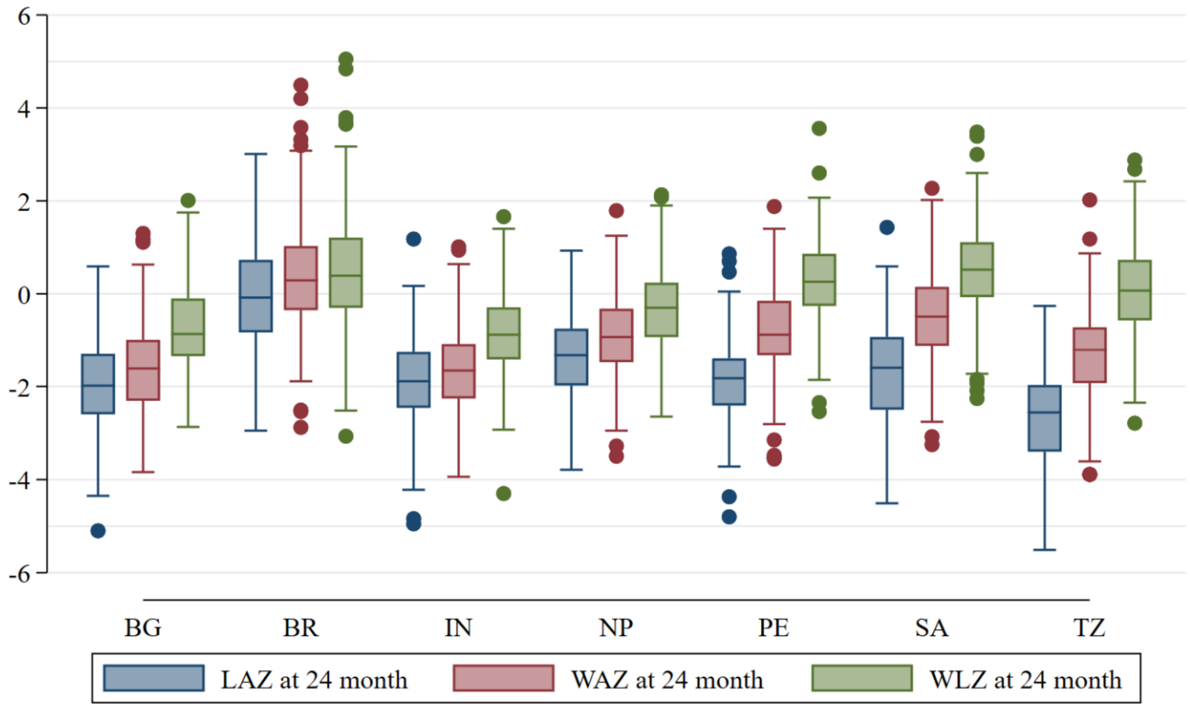

Supplementary Figure 2. Box-and-whisker plots are shown to visualize the LAZ, WAZ and WLZ at 24 month
